# Effectiveness of narrow vegetative filter strips in regulating sediment deposition in floodplain agricultural drainage ditches

**DOI:** 10.1002/jeq2.70280

**Published:** 2026-10-08

**Authors:** Xuechao Chen, Zhiming Qi, Birk Li, Monique Poulin, Shiv Prasher, Fan Zhang, Ziwei Li, Jiaxin Wang, Xiaoping Chen, Haomiao Cheng, Xizhi Nong

**Affiliations:** ^1^ Department of Bioresource Engineering McGill University Sainte‐Anne‐de‐Bellevue Quebec Canada; ^2^ Department of Phytology Université Laval Quebec City Quebec Canada; ^3^ State Key Laboratory of Water Disaster Prevention Hohai University Nanjing China; ^4^ Yangtze Institute for Conservation and Development Hohai University Nanjing China; ^5^ School of Environment Tsinghua University Beijing China; ^6^ College of Hydraulic Science and Engineering Yangzhou University Yangzhou China; ^7^ College of Civil Engineering and Architecture Guangxi University Nanning China

## Abstract

Floodplain agroecosystems are particularly susceptible to sediment redistribution due to seasonal flooding, snowmelt, and agricultural activities. However, field‐based evidence on the performance of narrow vegetative filter strips (VFS) under such dynamic hydrological conditions remains limited. This study evaluated the effectiveness of narrow VFS (2 and 4 m) in regulating sediment deposition within agricultural drainage ditches of the Lake Saint‐Pierre floodplain, Québec, Canada, a cold‐region agroecosystem characterized by recurrent spring flooding. From 2019 to 2023, sediment deposition dynamics were monitored across three floodplain sites using repeated total station surveys, complemented by UAV‐based LiDAR measurements. Annual sediment deposition volumes were quantified and standardized by ditch length (m^3^ m^−^^1^) to account for site‐scale variability, and statistical analyses were used to assess VFS effects across widths and over time. Across sites, 4‐m VFS consistently reduced sediment deposition by approximately 30% relative to control ditches without VFS (*p* < 0.10). In contrast, 2‐m VFS exhibited highly site‐dependent and statistically nonsignificant effects, reflecting interactions among VFS width, ditch morphology, and hydrological connectivity during flood and snowmelt periods. Differences in sediment deposition between growing and nongrowing seasons were modest, suggesting that sediment inputs are primarily controlled by episodic flood and snowmelt events rather than seasonal vegetation cover. Comparisons between LiDAR‐ and total station‐derived ditch volumes showed strong agreement, with an average difference of approximately 10%. These results demonstrate that narrow VFS can contribute to sediment regulation in floodplain agricultural drainage networks, although their effectiveness is constrained by hydrological context and strip width.

## Core Ideas

- Floodplain drainage ditches accumulate sediment mainly during snowmelt and flooding.
- Narrow vegetative filter strips can reduce sediment buildup in floodplain ditches.
- Four‐meter filter strips reduced sediment deposition by about 30% across sites.
- Two‐meter filter strips showed variable and site‐dependent performance.
- Flood‐driven hydrology limits the effectiveness of narrow buffer strips.

## Plain Language Summary

Floodplain farmland often experiences flooding and snowmelt that move soil into drainage ditches, reducing their performance. Vegetative filter strips (VFS) are narrow bands of plants used to slow water and trap sediment, but their effectiveness in flood‐prone areas is uncertain. This study examined 2‐m‐ and 4‐m‐wide VFS in the Lake Saint‐Pierre floodplain in Québec, Canada. Field measurements from 2019 to 2023 showed that 4‐m‐wide strips reduced sediment buildup in ditches by about 30%, while 2‐m‐wide strips produced weak and inconsistent results. Similar sediment levels across seasons suggest that flooding and snowmelt, rather than growing‐season conditions, control sediment movement. Wider filter strips, therefore, provide more reliable sediment reduction in floodplain settings.

**Abbreviations**
BDF: Baie‐du‐Febvre
STC: St. Cuthbert
VFS: vegetative filter strips
YAM: Yamachiche

## 1 INTRODUCTION

The St. Lawrence River is one of North America's largest river systems, with the second‐largest discharge on the continent after the Mississippi (Meybeck & Ragu, 1997), and it forms the world's largest fluvial estuary (Dinauer & Mucci, 2017). Along its course, the St. Lawrence Lowlands—particularly the Lake Saint‐Pierre (LSP) floodplain—are characterized by fertile soils, high biodiversity, and intensive agricultural activity (Moritz et al., 2015; Nicole, 2020; Nicole & Dauphin, 2019). Agriculture has long shaped this floodplain landscape, in which perennial vegetation has largely been replaced by annual crops such as corn and soybeans (Jobin & Brodeur, 2023). As a result, a dense network of agricultural drainage ditches now plays a central role in regulating surface water flow and sediment redistribution across the floodplain.

Seasonal flooding is a dominant hydrological feature of the LSP floodplain, driven primarily by spring snowmelt and ice‐jam dynamics in the St. Lawrence River (Buttle et al., 2016; Javelle et al., 2003; Magnan et al., 2022). During flood events, lowland fields and drainage ditches receive large sediment inputs through lateral inundation, overbank flow, and low‐velocity deposition processes (Van Maren et al., 2015; Walling & Fang, 2003). Unlike upland agricultural systems, where sediment delivery is mainly controlled by hillslope runoff, sediment redistribution in floodplains is often governed by hydrological connectivity between fields, ditches, and the river channel. As a consequence, agricultural drainage ditches act not only as conveyance structures but also as major sediment sinks within floodplain agroecosystems.

Vegetative filter strips (VFS) are widely promoted as best management practices to intercept surface runoff, reduce flow velocity, enhance infiltration, and trap sediment before it reaches surface water (Dosskey, 2001; Dosskey et al., 2002; Lee et al., 2003; Muñoz‐Carpena et al., 1999; Syversen & Borch, 2005). Numerous studies conducted in upland or non‐flooded agricultural settings have reported substantial reductions in sediment transport across a range of spatial scales, from plot‐based experiments to small catchments (Arora et al., 2003; Borin et al., 2005; Bouldin et al., 2004; Duchemin & Hogue, 2009; Hussein et al., 2007; Patty et al., 1997; Saleh et al., 2018). However, the effectiveness of VFS is strongly influenced by hydrological context. Concentrated flow paths, channelized drainage networks, and seasonal hydrological variability can substantially reduce sediment trapping efficiency (Arora et al., 2010; Dodd & Sharpley, 2016; Dorioz et al., 2006; Hay et al., 2006; Hösl et al., 2012; Hénault‐Ethier et al., 2017; Hickey & Doran, 2004; Verstraeten et al., 2006). In floodplain agroecosystems, these limitations may be further amplified by recurrent overbank flooding and snowmelt‐driven lateral inundation, which can bypass conventional runoff‐interception mechanisms and alter sediment transport pathways.

Despite their practical relevance, empirical evidence on the performance of narrow VFS (1–10 m)—the widths most feasible for implementation on working farms—remains limited, particularly in flood‐dominated landscapes (Anebagilu et al., 2021; Hickey & Doran, 2004). Existing studies rarely address how narrow VFS function within extensive drainage‐ditch networks subject to periodic flooding, where sediment redistribution is controlled by interactions among strip width, ditch morphology, vegetation continuity, and hydrological connectivity. As a result, guidance on the design and expected performance of narrow VFS in floodplain agricultural systems remains highly uncertain.

The objective of this study was to evaluate whether narrow VFS can effectively regulate sediment deposition within agricultural drainage ditches under flood‐dominated conditions. Focusing on the LSP floodplain, a cold‐region agroecosystem subject to recurrent spring flooding and snowmelt‐driven sediment redistribution, we addressed two specific objectives: (i) to quantify how VFS width (0, 2, and 4 m) influences sediment deposition dynamics in drainage ditches across multiple floodplain sites using repeated geomorphic surveys, and (ii) to assess the consistency of sediment volume estimates derived from traditional total station surveys and UAV‐based LiDAR measurements for monitoring ditch‐scale sediment accumulation.

## 2 MATERIALS AND METHODS

### 2.1 Experimental sites and design

The study was carried out at three experimental sites located on the Lac Saint‐Pierre floodplain, Québec, Canada, near St. Cuthbert (STC—46°07′48.0″ N, 73°07′26.4″ W), Yamachiche (YAM—46°16′04.8″ N, 72°51′50.4″ W), and Baie‐du‐Febvre (BDF—46°08′34.8″ N, 72°42′46.8″ W) (Figure 1). The experimental sites represent a typical agricultural operation in the coastal zone of LSP, with an annual corn–soybean rotation, fall tillage, and drainage via open ditches directly to the lake. The average annual temperature in the region is approximately 7°C, and the average annual precipitation is approximately 900 mm (Environment and Climate Change Canada [ECCC], 2023). All ditches at these sites are regularly exposed to risks of sediment accumulation and seasonal flooding.

**FIGURE 1:**
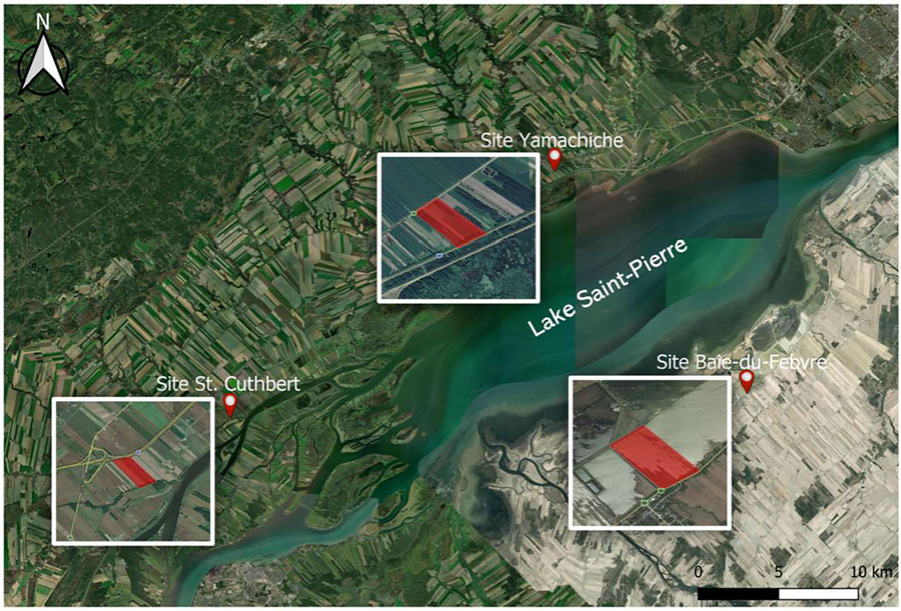
Location of the three experiment sites.

This field experiment employed a randomized complete block design, with each site (agricultural ditch location) serving as a block. Within each block, three treatments were implemented: (1) Control with no VFS (0 m), (2) 2‐m VFS, and (3) 4‐m VFS, all established along both sides of the ditch. Prior to the installation of VFS, all nine selected ditches were dredged in November 2019 to create a uniform initial condition across sites, similar to the standardized pre‐seeding field preparation described by Damar et al. (2025) for perennial strip establishment around LSP. Following dredging, VFS treatment were assigned randomly within each block to minimize the effect of local environmental heterogeneity. This layout ensured that treatment effects on sediment deposition and ditch infilling could be compared within and across blocks while accounting for spatial variability in hydrology, soil texture, and flooding regime.

In spring 2020, 12 reed canary grass (RCG) (*Phalaris arundinacea*)–oat (*Avena sativa*) mixture VFS, ranging from 600 to 1000 m long, were planted on both sides of the 2‐m and 4‐m VFS ditches (Figure S1). RCG (*P. arundinacea*) was chosen for its ability to establish under the highly flooded and clay‐rich conditions of the LSP floodplain and to form dense, persistent vegetation suitable for fish spawning. Oat (*A. sativa*) was included as a short‐lived companion crop to reduce weed pressure and improve early RCG establishment, while naturally winterkilling to avoid long‐term competition (Damar et al., 2025). They were all seeded with a grain drill equipped with either a mile box or a ridge seeder at a rate of 29 and 71 kg ha^−1^, respectively, without the application of fertilizer. All strip portions that showed failed or poor RCG establishment (approximately 60% of total strip length) were completely reseeded or overseeded in May 2021. All strips were mowed between August 1 and August 15 every year to prevent the regrowth of companion crops and weeds.

**Core Ideas**

- Floodplain drainage ditches accumulate sediment mainly during snowmelt and flooding.
- Narrow vegetative filter strips can reduce sediment buildup in floodplain ditches.
- Four‐meter filter strips reduced sediment deposition by about 30% across sites.
- Two‐meter filter strips showed variable and site‐dependent performance.
- Flood‐driven hydrology limits the effectiveness of narrow buffer strips.

### 2.2 Total station surveys

#### 2.2.1 Ditch cross‐section measurements

A series of total station surveys has been conducted in the study area since 2019, and the morphology of the ditches was characterized by measuring their cross‐sections at 20 m intervals. In total, seven total station geological surveys were performed to measure the cross‐section profiles of the ditches: once after the ditch trenching in 2019, twice per year in 2020 and 2021 (at both the start of the growing and nongrowing season), and once per year (in June) in 2022 and 2023. However, the first geological survey was incomplete due to severe winter weather in late 2019, and the VFS were replanted due to poor growth in spring 2021.

The reflective stakes were set at 20 m intervals on both sides of the ditch to mark the cross‐sections to be repeatedly measured. The ditch cross‐section profiles’ topography was measured using a Leica TS‐06 manual total station and its associated GPR‐1 prisms. The reference points were all established against fixed objectives, such as bolts at the base of the streetlight and marking points for drainage pipes. The cross‐section topography was constructed via a one‐dimensional geodetic measurement (elevation only) at 25 cm intervals along the cross‐section profile (20 measurement points per cross‐section). To prevent the survey operation's disturbance to the ditch geomorphology, a modified aluminum ladder, graduated every 25 cm, was placed above the ditch as a stepping platform.

#### 2.2.2 Ditch volume and sediment deposition calculation

The definition of sampled ditch cross‐section area (𝐴) is based on a slight modification of Roelens et al. (2016). The area between the cross‐section profile sides and its highest elevation's horizontal projection line was set to be the cross‐section area (Figure 2). The horizontal projection line position was altered from the lowest profile elevation maxima to the profile's highest elevation. This accommodates the interest in monitoring topographic change on both ditch sides.

**FIGURE 2:**
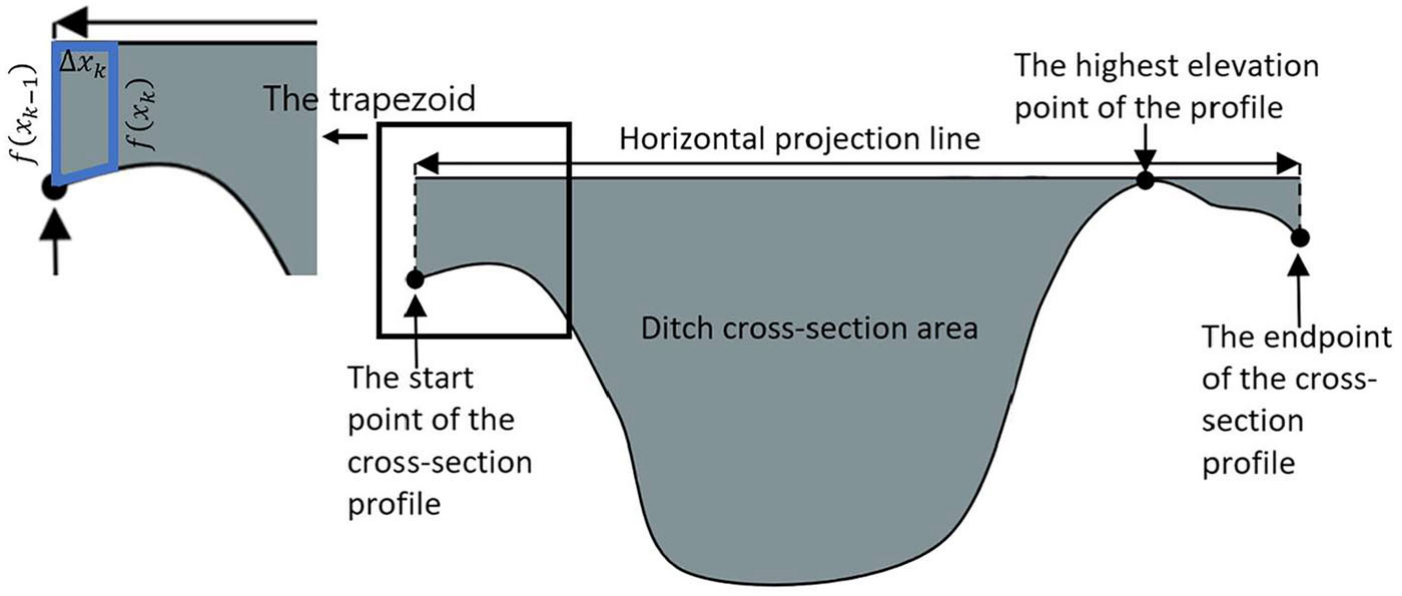
Cross‐sectional area definition.

The area below the horizontal projection was the cross‐sectional area of the ditch. To calculate the cross‐sectional area (A), the trapezoidal rule was used (Figure 2). Since the grid spacing was nonuniform, the cross‐sectional area was calculated based on Equation (1):

(1) $$A=\int_{a}^{b}f\left(x\right)\mathrm{dx}=\sum_{k=1}^{n}\frac{f\left(x_{k-1}\right)+f\left(x_{k}\right)}{2}\Delta x_{k}$$

where A is ditch cross‐section area (m^2^), a is the start point of each cross‐section profile, b is the endpoint of each cross‐section profile, $x_{k}$ is the horizontal distance of each measured point from the start points of each profile (m), f(x) is the vertical distance of each measured point from the highest elevation points of each cross‐section profile (m), k is the $k_{\mathrm{th}}$ of the measured point for each cross‐section profile, n is the number of measured points surveyed for each ditch cross‐section profile, and $\Delta x_{k}$ = $x_{k}-x_{k-1}$ (m).

The ditch's topographic profile was sampled as cross‐sections at 20 m intervals (the number of cross‐sections sampled varies by the length of each ditch). Individual ditch's volume (V) was estimated by following Equations (2) and (3):

(2) $$A_{K}=\frac{\left(A_{i}+A_{i+1}\right)}{2}$$

(3) $$V=\sum_{K=1}^{N-1}A_{K}\times \;\mathrm{interval}$$

where $A_{K}$ is the average area of two adjacent surveyed cross‐section profiles (m^2^), $A_{i}$ is the area of the surveyed cross‐section profile (m^2^), K is the Kth of the calculated cross‐section profile for the ditch, i is the ith of the surveyed cross‐section profile for the ditch, V is ditch volume measured (m^3^), and N is the number of ditch cross‐section profiles surveyed for the ditch.

The volume change rates for ditch and sediment deposition (V%) were both calculated by Equation (4):

(4) $$V\%\;=\frac{\left(V_{m\;-\;1}-V_{m}\right)}{V_{m\;-\;1}}\;\times \;100\%$$

where V% is the volume change rate, V is the ditch volume measured (m^3^), and m is the $m_{\mathrm{th}}$ of the total station measurement. Sediment deposition was directly shown by the volume of sediment deposits in this study.

### 2.3 LiDAR aerial surveys

#### 2.3.1 Data acquisition

All studied ditches have been scanned four times (November 2019 (before trenching), December 2019 (after trenching), October 2020, and October 2021), using the YellowScan Surveyor LiDAR system accompanied by a DJI Matrice 600 UAV (Figure S2a), with the LiDAR system's in situ GNSS antenna connected to a Spectra SP80 GNSS receiver (Figure S2b) in the base‐station mode. The UAV was kept within a 2 km radius of the GNSS base station and recorded the LiDAR sensor position using the post‐processed kinematic positioning method.

The LiDAR system has a 903 nm laser wavelength and can generate two echoes per shot. The scanning angle was set to 40° (±20°). The flight height was controlled to be 15 m above the ground. The collected 3D point cloud data have a point density of 250–400 points m^−2^. These point cloud data were processed using the YellowScan Cloud Station software associated with the LiDAR sensor for noise filtering and point cloud classification based on echo order and signal return strength. The processed point cloud data were used to generate a digital terrain model with a spatial resolution of 5 cm based on all the bare‐earth‐classified data points.

#### 2.3.2 Data processing algorithms

After collecting raw data in the field (Figure S3), the data were further processed and analyzed in the laboratory. MATLAB R2024a (MathWorks) was used for data processing and volume calculations, yielding detailed analyses and accurate results.

All ditches' 3D models were reconstructed from bare land points (LiDAR classification 2) and GPS data to remove other points, primarily low vegetation and water. Figure 3A shows an example of one drainage ditch's bare ground point cloud. Low vegetation is defined as vegetation with a canopy height of less than 30 cm, medium vegetation is defined as vegetation with a canopy height ranging from 30 to 250 cm, and that with a canopy height larger than 250 cm is referred to as high vegetation (Wang et al., 2017). Due to the dense low vegetation covering the ditch bank and water in the ditch, the density of the laser point clouds cannot be uniform. Especially at the bottom of the ditch, the density is too small, and the points are sparse (Figure 3B). To avoid the above problem, each ditch was cut into 0.5 m long segments according to the ditch direction (the ditch sides opposite the drainage flow were defined as upstream in this study). A representative cross‐section would be selected for each segment.

**FIGURE 3:**
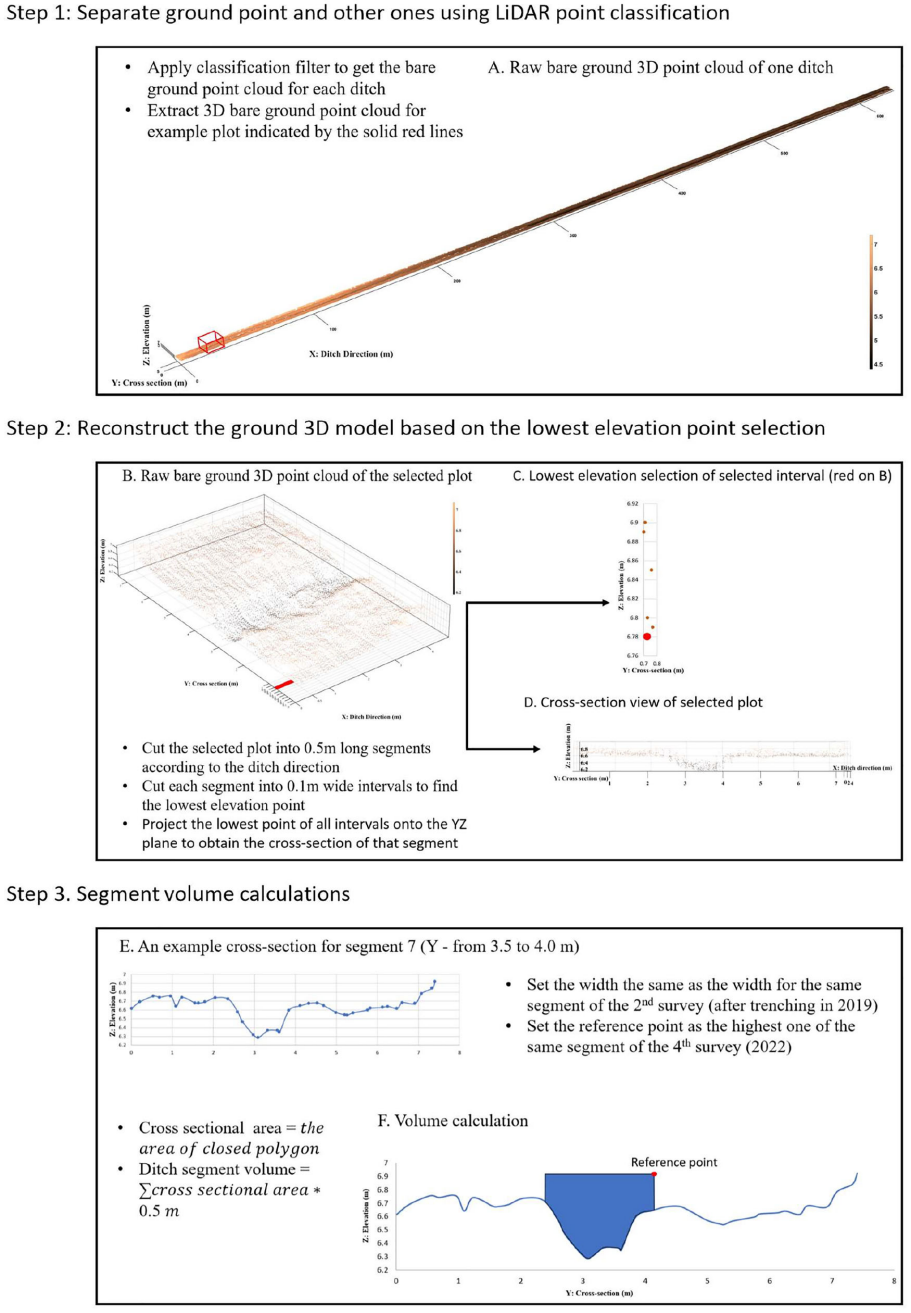
Data processing pipeline. (A) an example of the bare ground point cloud of one ditch, (B) the raw bare ground point cloud of the selected plot, (C) an example of the lowest point selection for one interval, (D) the cross‐section view after point selection, (E) a cross‐section example, and (F) volume calculation.

According to the cross‐section axis, each segment was cut into 0.1 m‐wide intervals to find the lowest elevation point (Figure 3C, D), which would be used for the volume calculation for the relevant segment. The projection of all intervals’ lowest point onto the YZ plane is defined as the cross‐section of that segment (Figure 3E). The width for all four time measurements is set to match the width used for the second survey (after trenching in 2019). The area below the level of the reference point, which is the highest elevation point of the same segments of the fourth survey (2022), was the cross‐sectional area of the segment (Figure 3F). To calculate the cross‐sectional area (A), the trapezoidal rule was used (Figure 2). Since the grid spacing was nonuniform, the cross‐sectional area was calculated similarly to Equation (1), as shown below (Equation 5):

(5) $$A=\int_{a}^{b}f\left(x\right)\mathrm{dx}=\sum_{k=1}^{n}\frac{f\left(x_{k-1}\right)+f\left(x_{k}\right)}{2}\Delta x_{k}$$

where a is the start lowest elevation point of each segment cross‐section profile, b is the lowest elevation point of each cross‐section profile, $x_{k}$ is the horizontal distance of each ground point from the start point of each profile (m), $\Delta x_{k}$ is the horizontal distance between two adjacent ground points, and f(x) is the vertical distance of each ground point from the reference point of each cross‐section profile (m).

Based on each segment's cross‐sectional area and the segment length (0.5 m), the individual ditch's volume (V) was estimated by following Equation (6):

(6) $$V=\sum_{i=1}^{N-1}A_{i}\times \;\mathrm{interval}$$

where $A_{i}$ is the area of the segment cross‐section profiles (m^2^), i is the *i*th segment cross‐section profile for the ditch, N is the number of ditch cross‐section profiles surveyed for the ditch, and the interval is 0.5 m.

### 2.4 Statistical analysis

IBM SPSS Statistics software (Version 28.0.0.0) was used for the statistical analysis. Sediment deposition volumes (m^3^) were measured annually at three experimental sites (YAM, STC, and BDF) under three VFS width treatments (0, 2, and 4 m). To account for differences in ditch length among sites, all volume values were standardized to sediment deposition per unit length (m^3^ m^−1^). A one‐way analysis of variance (ANOVA) was performed, with standardized sediment deposition data collected from June 2020 to June 2023 as the response variable. The factor “VFS width” comprised three levels (0, 2, and 4 m), with each level including three replicates from the three sites (YAM, STC, and BDF). Tukey's honestly significant difference (HSD) test was used for pairwise comparisons among treatments (e.g., 0 vs. 2 m) (Kim, 2015). Finally, a two‐way repeated‐measures ANOVA was employed to examine how VFS efficiency changed over time, treating measurement time as the within‐subject factor and VFS width as the between‐subject factor (Dien & Santuzzi, 2005). All significance tests were evaluated at *p* = 0.10.

## 3 RESULTS AND DISCUSSION

### 3.1 Total station surveys

Results by total station surveys showed that VFS reduced sediment by an average of 21.4% with a 2‐m width and 29.2% with a 4‐m width, with no significant difference between the two treatments. Sediment deposition also varied with annual rainfall and ditch length, with some areas experiencing erosion.

#### 3.1.1 Ditch volumes under control and different treatments

Volume data collected at different times indicated that all ditches at the experimental sites experienced sediment deposition during the experimental period (Table 1). The deposition volume of sediment in the ditches ranges from 152.5 to 419.4 m^3^ over all years. The maximum sediment deposition happened in the ditch without VFS at STC, and the minimum occurred in the ditch with 4‐m VFS at YAM. Based on the one‐way ANOVA results (*p* > 0.10), there was no statistically significant difference in ditch sediment deposition among the three VFS widths (0, 2, and 4 m) from June 2020 to June 2023 within the scope of this experiment. There was also no statistically significant interaction (*p* > 0.10) between the factor “time” and the factor “VFS width” after analyzing all ditch volumes at different measurements by a two‐way repeated measures ANOVA, showing VFS efficiency has not changed significantly over time in the littoral zone of LSP.

**TABLE 1:** Ditch volume during the experimental period.

Site name	VFS width (m)	Ditch volume (m^3^)					Sediment deposition (2020.6–2023.6) (m^3^)
		2020.6	2021.6	2021.10	2022.6	2023.6	
YAM	0	1016.6	911.8	854.2	794.7	749.5	267.1
	2	767.8	680.4	608.6	515.5	486.1	281.7
	4	1162.2	1004.7	956.3	951.7	1009.7	152.5
STC	0	1121.8	1024.3	912.7	818.8	702.3	419.4
	2	1046.7	958.7	870.0	810.3	726.3	320.4
	4	896.0	811.8	747.7	673.8	585.2	310.8
BDF	0	Insufficient^a^	1339.3	1254.2	1157.6	1020.9	318.4
	2	Profile	1129.1	1042.6	999.2	920.3	208.9
	4	Numbers	1318.3	1279.6	1238.3	1078.3	240.0

Abbreviations: VFS, vegetative filter strips; YAM, Yamachiche; STC, St. Cuthbert; BDF, Baie‐du‐Febvre.

^a^ There are not enough profile numbers to determine the ditch volume because only the measurement for the first 580 m of the 1000‐m‐long ditches was completed at BDF in June 2020.

The yearly sediment deposition volumes for each ditch varied (Table S1). The prominent sediment deposition in the ditches occurred from June 2021 to June 2022. The average deposition in June 2021–2022 was 135.38 m^3^, compared to 103.25 m^3^ in June 2020– 2021 and 75.7 m^3^ in June 2022–2023. The total precipitation from July 2021 to June 2022 was 899.3 mm, the driest compared to 1068.2 and 1082.6 mm in the same months of 2020–2021 and 2022–2023, respectively, at the Pierreville weather station (46°05′ N, 72°50′ W). Probably in a drier year, less sediment in the ditches was washed down to the lake. The field sedimentation data exhibited substantial variability and inconsistency. For example, the 2‐m VFS showed contradictory effects at three sites—slightly increased sediment deposition at YAM and reduced it at STC and BDF—which may be caused by the poor growth of the 2‐m VFS at YAM. The 4‐m VFS ditch at the YAM site witnessed the highest sediment deposition among all three ditches in June 2020–June 2021, but the reason is unknown. These interannual patterns are illustrated in Figure 4.

**FIGURE 4:**
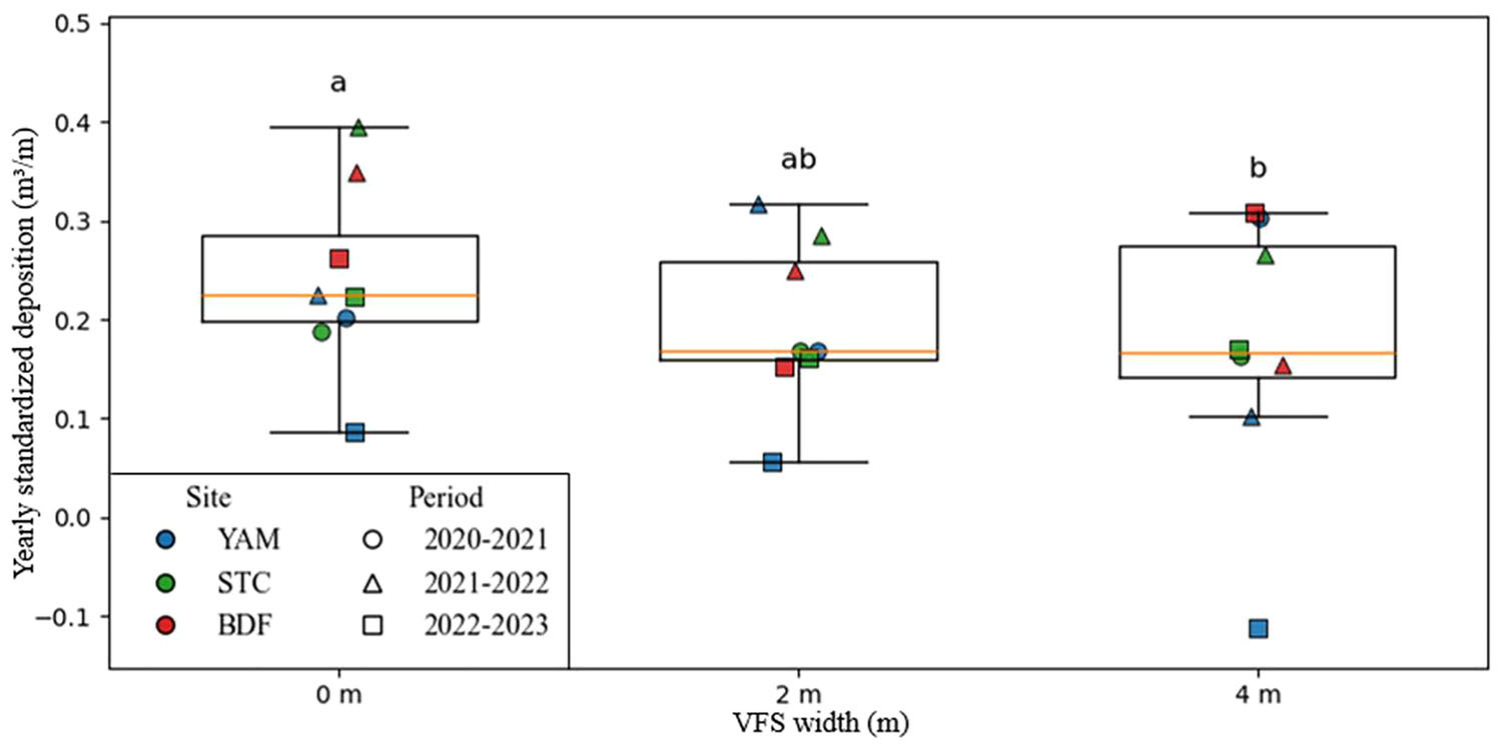
Standardized sediment deposition across VFS widths showing site‐ and year‐level variability.

Based on the yearly sediment deposition volumes collected from June 2020 to June 2023, a one‐way ANOVA indicated that VFS width had a significant effect on sediment deposition per unit ditch length (*p* = 0.06) (Figure 4; Table S2). Tukey's post hoc test revealed that 4‐m VFS ditches had significantly lower sediment deposition than control ditches without VFS (*p* < 0.10), whereas the 2‐m treatment was not significantly different from either 0 or 4 m, indicating an intermediate effect. On average, the annual sediment deposition volumes in 2‐m and 4‐m VFS ditches were 101.7 and 91.5 m^3^, corresponding to reductions of 21.4% and 29.2%, respectively, compared to control ditches (Table S1).

#### 3.1.2 Sediment accumulation during growing and nongrowing season period

Sediment deposition volumes were separated into two periods: the growing season (June–October 2021) and the nongrowing season (October 2021–June 2022). There was no significant difference (*p* > 0.10) between sediment deposition volumes during the two periods when the VFS widths were 0, 2, and 4 m, by paired‐sample *t*‐tests. Based on the data collected from June 2021, October 2021, and June 2022, sediment deposition volumes during the growing season (2021.6–2021.10) and nongrowing season period (2021.10–2022.6) (Table S3) were taken into comparison. There is little difference (*p* > 0.10) in the sediment deposition volumes between the two periods as no‐VFS, ranging from −15.7% to 13.4%. However, as the VFS width increases, the difference gets more prominent, and the deposition situations for the ditches vary. There are three ditches with decreasing deposition volume during the nongrowing season, ranging from −90.5% to −32.6%. The deposition volume in the other three ditches showed an increasing trend of 29.9%, 15.3%, and 6.7%, respectively.

When averaged across both periods, mean sediment deposition differed among VFS widths. A one‐way ANOVA indicated a marginal treatment effect (*p* = 0.102). Tukey's HSD test revealed that 4‐m VFS ditches had significantly lower mean sediment deposition compared with 0‐m and 2‐m ditches (*p* < 0.10), while no difference was detected between 0‐m and 2‐m treatments. On average, yearly sediment deposition volumes were 84.8 m^3^ for 0 m, 82.3 m^3^ for 2 m, and 50.4 m^3^ for 4 m (Table S3).

### 3.2 LiDAR surveys

#### 3.2.1 Ditch volumes under control and different treatments by LiDAR surveys

Based on the LiDAR data from the last three surveys, ditch volumes were determined for comparison, considering time trends (Table S4). At the YAM site, the ditch volume for VFS width of 0 m increased from 1025.869 m^3^ in 2019 to 1377.816 m^3^ in 2021. In contrast, the volumes for VFS widths of 2 and 4 m showed a decreasing trend over the same period. At the STC site, the ditch volume for a VFS width of 0 m slightly increased, while the volumes for widths of 2 and 4 m exhibited variability but generally decreased from 2019 to 2021. The BDF site consistently showed the highest initial ditch volumes, which then decreased over time for all VFS widths. Overall, most sites demonstrate a general trend of decreasing ditch volumes from 2019 to 2021, indicating natural sedimentation processes, consistent with total station survey results. Due to position changes following the trenching of the ditch with 0‐m VFS at YAM, the first LiDAR survey results were not used for ditch volume comparison.

#### 3.2.2 Volume comparison

The volumes of all nine ditches, determined by total station and LiDAR surveys conducted in October 2021, were compared using consistent width and length measurements (Table 2). The average width measurements between LiDAR and total station surveys are highly consistent across all sites and VFS widths. The overall average widths are 2.29 m for LiDAR surveys and 2.30 m for total station surveys, demonstrating a strong agreement between the two methods. However, there is some variability in volume measurements, with differences ranging from −6.64% to 27.26% depending on the site and VFS width. The overall average volumes are 688.61 m^3^ for LiDAR surveys and 605.27 m^3^ for total station surveys, resulting in an average difference of 9.46%. The relatively low average percentage difference of 9.46% and no significant difference between them (*p* > 0.10) indicate that LiDAR is reasonably accurate compared to total station measurements, although specific conditions or ditch characteristics might influence the accuracy.

**TABLE 2:** Comparison of ditch volumes measured by LiDAR and total station surveys (October 2021).

Site name	VFS width (m)	Ditch length (m)	LiDAR surveys		Total station surveys		Different %
			Average width (m)	Volume (m^3^)	Average width (m)	Volume (m^3^)	
YAM	0	540	2.20	591.42	2.22	461.40	21.98
	2	560	2.25	300.22	2.26	320.16	−6.64
	4	540	2.54	482.79	2.54	487.56	−0.99
STC	0	800	1.86	290.53	1.87	277.10	4.62
	2	800	2.04	427.82	2.01	430.09	−0.53
	4	800	2.63	745.43	2.61	582.79	21.82
BDF	0	780	2.53	975.25	2.55	709.37	27.26
	2	940	2.39	1216.10	2.44	1223.96	−0.65%
	4	980	2.20	1167.92	2.23	955.00	18.23%
Average			2.29	688.61	2.30	605.27	9.46%

Abbreviations: VFS, vegetative filter strips; YAM, Yamachiche; STC, St. Cuthbert; BDF, Baie‐du‐Febvre.

#### 3.2.3 Applicability and limitations of LiDAR‐based and total station–based volume estimates

Comparisons between UAV‐based LiDAR and total station surveys showed strong overall agreement in ditch volume estimates, with an average difference of approximately 9%–10%. This level of consistency indicates that UAV‐based LiDAR is a viable tool for quantifying sediment accumulation in agricultural drainage ditches at the floodplain scale, particularly when repeated measurements over large areas are required. The close agreement in average ditch widths derived from both methods further supports the reliability of LiDAR for capturing overall ditch geometry.

Despite this general consistency, volume differences varied substantially among sites and VFS treatment, with discrepancies exceeding 20% in several cases. These differences highlight important methodological and environmental constraints associated with LiDAR‐based measurements in drainage‐ditch environments. Dense vegetation, standing water, and complex ditch geometries can reduce point‐cloud density near ditch bottoms, leading to uncertainty in volume reconstruction. In contrast, total station surveys, which rely on direct ground‐based cross‐sectional measurements, are less sensitive to vegetation and water cover but require substantially greater labor and time investment.

Differences between methods were further amplified when volume estimates were used to project time to ditch blockage under a linear siltation assumption. LiDAR‐based projections tended to indicate shorter timeframes to partial or complete blockage, reflecting the method's sensitivity to fine‐scale elevation changes across the ditch surface. Total station–based projections, by contrast, emphasized longer term trends by smoothing localized variability across fewer measurement points. These contrasting behaviors suggest that LiDAR may provide more conservative estimates of maintenance needs, whereas total station surveys may better capture long‐term average sedimentation rates.

Importantly, neither method fully resolves the complex, nonlinear nature of sediment accumulation in floodplain drainage ditches, where episodic flood events, bank failure, and vegetation dynamics interact. As such, time‐to‐blockage estimates derived from linear extrapolation should be interpreted as indicative rather than predictive. A combined monitoring strategy, using UAV‐based LiDAR for rapid spatial assessment and total station surveys for detailed long‐term tracking, may offer the most robust approach for managing sediment accumulation in floodplain agricultural drainage networks.

### 3.3 Estimated ditch maintenance time by two surveys

Based on the sedimentation results obtained by the two measurement methods, we estimated the time when each ditch would reach 60%, 70%, 80%, 90%, and 100% blockage. This estimation was used to assess potential ditch maintenance needs. The estimated time (in years) by total station surveys represents the period from each ditch's earliest measurement until it reaches the specified blockage levels, under the assumption of linear siltation (Table S5). The initial volumes in October 2021 in Table 1 were treated as 100% usable capacity.

From the predicted blockage years for YAM, STC, and BDF under 0, 2, and 4 m VFS widths at various blockage levels (60%, 70%, 80%, 90%, and 100%), increasing VFS width tends to effectively delay the progression of culvert/drainage blockage—especially in postponing complete (100%) blockage. Exceptions (e.g., YAM at 2 m) underscore that in certain locations, smaller VFS widths might trigger unfavorable hydraulic or sediment transport effects, leading to earlier blockage. Table S6 shows the estimated years (starting from November 2019) for each ditch to reach 60%, 70%, 80%, 90%, and 100% blockage as inferred from LiDAR surveys. These estimates were based on a linear siltation assumption, calculated from the volume differences between November 2019 and October 2021. However, for the ditch at YAM with a 0‐m treatment, the ditch volume increased from November 2019 to October 2021. As a result, the linear siltation model could not be applied to estimate the blockage time for this case.

At STC and BDF, wider VFS (2 or 4 m) consistently help delay the progression from partial (60%, 70%, 80%, and 90%) to full (100%) blockage, compared to having no VFS. At YAM, both 2 and 4 m produce the same timeline, suggesting minimal difference in sediment interception effectiveness under LiDAR‐based estimation at these widths.

In practice, both methods are reliable for volume estimation (as shown by the average 9.46% difference in Table 2), but when used for time‐to‐blockage projections, the LiDAR method emphasizes short‐term variability, whereas the total station method emphasizes long‐term trends. LiDAR surveys capture very fine‐scale elevation and sediment changes across the entire ditch surface. When these high‐resolution changes are projected forward, even small variations can result in shorter predicted blockage times, making LiDAR appear more “conservative” in estimating ditch lifespan. Total station surveys, by contrast, rely on a limited number of cross‐sectional points. This tends to smooth out localized sedimentation, leading to a slower predicted rate of blockage accumulation and thus longer blockage times.

### 3.4 Effectiveness of VFS under flood‐dominated conditions

Results from this study demonstrate that VFS can reduce sediment deposition in agricultural drainage ditches within the LSP floodplain, but their effectiveness is strongly constrained by hydrological context and strip width. Across the three study sites, 4‐m‐wide VFS consistently reduced sediment deposition relative to control ditches, whereas 2‐m‐wide strips exhibited variable and site‐dependent performance. This contrast suggests that VFS effectiveness in floodplain agroecosystems is governed not solely by vegetation presence, but by whether strip width exceeds a minimum functional threshold under flood‐dominated hydrological regimes.

The stable performance of 4‐m VFS across sites indicates that this width is sufficient to influence sediment redistribution processes despite recurrent spring flooding and snowmelt‐driven inundation. Wider strips likely provide greater surface roughness, longer flow paths, and more continuous vegetation cover, enhancing their capacity to attenuate flow velocity and promote sediment settling even when lateral inundation occurs. In contrast, 2‐m VFS appear more vulnerable to hydraulic bypassing during high‐water events, particularly where ditch morphology, local slope, or vegetation discontinuity reduces interception efficiency. This interpretation is consistent with flume and field studies showing that sediment trapping efficiency decreases sharply under concentrated or high‐energy flow conditions and that narrow buffers are especially sensitive to slope and flow convergence (Gall et al., 2018; Jin & Römkens, 2001; Wu et al., 2023).

Site‐specific responses observed for the 2‐m VFS further highlight the role of local controls. At the YAM site, higher ditch slope gradients and partial vegetation failure along the strip coincided with increased sediment deposition relative to the control, suggesting that insufficient strip width can amplify, rather than mitigate, sediment accumulation under unfavorable hydraulic conditions. Such responses are not anomalies but reflect the complex interaction between VFS width, ditch geometry, and flood‐driven sediment transport pathways in floodplain environments.

The lack of a significant interaction between time and VFS width indicates that VFS performance did not systematically degrade or improve over the monitoring period. Instead, interannual variability in sediment deposition was primarily driven by hydrological forcing, particularly the timing and magnitude of flood and snowmelt events. These findings contrast with upland systems, where VFS efficiency often increases with vegetation maturity and seasonal cover, and emphasize that in floodplain agroecosystems, episodic hydrological events can override vegetation‐driven controls.

Overall, the results suggest that narrow VFS function differently in flood‐dominated landscapes than in upland agricultural settings. Rather than providing gradual, incremental improvements with increasing width, VFS effectiveness in floodplain drainage networks appears to exhibit threshold behavior. Below this threshold, performance is highly variable and strongly site‐dependent; above it, sediment deposition is more consistently reduced. This distinction has important implications for the design and evaluation of land‐efficient buffer‐based management practices in floodplain agroecosystems.

### 3.5 External factors affecting VFS performance

Unlike the hydrologically regulated leaching plot experiments in the literature review, in the actual application of VFS in farmland, the ditch will be subject to artificial damage, such as ditch bank collapse caused by tractors. Artificial damage may cause errors in the measured cross‐section. The collapse of the ditch bank can cause the area of the cross‐section to increase, and otters nesting in the ditch can make the area smaller. One of the main difficulties encountered during the experimental period was maintaining the VFS. Several environmental factors can affect the establishment of RCG in VFS, particularly soil properties and rainfall timing. The growth of VFS cannot be guaranteed. For example, the second half of a 2‐m VFS at YAM grew poorly in 2023. Another point worth noting is that in actual agricultural drainage ditches, even the control ones without VFS, there is still a lot of vegetation in the ditches (Figure S4).

In‐ditch vegetation growth could affect the performance of the VFS in littoral zones, particularly during agricultural drainage periods (Cooper et al., 2004). The seasonal growth of aquatic vegetation in ditches can increase their hydraulic roughness by an order of magnitude, thereby dramatically affecting sediment‐trap efficiency (Watson, 1987). Hence, the sediment‐trap efficiencies of VFS can be linked to in‐ditch vegetation density in future studies. Moreover, beavers were found nesting upstream of the 2‐m VFS ditch at BDF in 2022 and 2023 (Figure S5), which may have influenced the trend of ditch volume change rate versus ditch length.

## 4 CONCLUSIONS

In this study, VFS has been shown to reduce sediment deposition in the agricultural drainage ditch in the littoral zone of LSP. During the experimental period, the 2‐m VFS showed standardized sediment removal rates of −5.8%, 23.8%, and 34.33%, respectively, relative to the control. The ditch with this type of VFS at YAM had higher sediment deposition (0.1 m^3^ m^−1^ per year). For STC and BDF, 2‐m VFS reduced sediment deposits by 0.064 and 0.11 m^3^ m^−1^ per year (*p* < 0.10). Moreover, the 4‐m VFS reduced sediment deposition by 42.7%, 26.0%, and 24.5% compared with the control (*p* < 0.10). The 4‐m VFS at three sites showed similar removal rates, decreasing by 0.073, 0.070, and 0.075 m^3^ m^−1^ per year. Moreover, as the VFS width increased, the average reduction in sediment deposition became more prominent. The 4‐m VFS reduced sediment deposits by 13.7% more than the 2‐m ones. However, VFS did not perform well in preventing sediment deposition during nongrowing seasons (*p* > 0.10). Three ditches with VFS exhibited a decrease in deposition volume during the nongrowing seasons compared to the deposition volume during the growing season, at an average of 32.7%. In contrast, three ditches with VFS showed an increasing trend in deposition volume during the nongrowing seasons, by 6.7%, 13.4%, and 15.3%, respectively. In future studies, in‐ditch vegetation density can be considered a factor affecting VFS performance, particularly during the growing season. Moreover, both LiDAR and total station surveys provide valuable tools for measuring ditch volumes, with LiDAR generally demonstrating strong accuracy and consistency. The variability in performance across sites highlights the need to carefully consider site characteristics and methodological limitations. Future work could focus on identifying the factors that affect LiDAR accuracy at challenging sites and on exploring ways to enhance its reliability across various conditions. The study also highlights the importance of ongoing monitoring and further research to better understand the long‐term effects of VFS. It is also important to address the practical challenges associated with their implementation in real‐world agricultural settings, given the considerable land area and the required vegetation management.

## AUTHOR CONTRIBUTIONS

**Xuechao Chen**: Conceptualization; data curation; formal analysis; funding acquisition; investigation; methodology; project administration; resources; software; supervision; validation; visualization; writing—original draft; writing—review and editing. **Zhiming Qi**: Conceptualization; data curation; formal analysis; funding acquisition; investigation; methodology; project administration; resources; software; supervision; validation; visualization; writing—original draft; writing—review and editing. **Birk Li**: Conceptualization; data curation; investigation; methodology; validation; writing—original draft. **Monique Poulin**: Conceptualization; investigation; project administration; resources; supervision; writing—original draft. **Shiv Prasher**: Conceptualization; formal analysis; methodology; writing—original draft. **Fan Zhang**: Conceptualization; data curation; investigation; project administration. **Ziwei Li**: Conceptualization; data curation; formal analysis; methodology; writing—original draft. **Jiaxin Wang**: Conceptualization; data curation; formal analysis; investigation; writing—original draft. **Xiaoping Chen**: Data curation; methodology. **Haomiao Chen**: Conceptualization; investigation. **Xizhi Nong**: Data curation; formal analysis; writing—original draft.

## CONFLICT OF INTEREST STATEMENT

The authors declare no conflicts of interest.

## Supporting information



The Supplemental Material provides detailed datasets and additional evidence supporting the main findings of this study. It includes yearly sediment deposition volumes (Table S1) and standardized deposition per ditch length (Table S2), highlighting treatment differences and interannual variability across sites. Seasonal deposition dynamics during growing and non‐growing periods are presented in Table S3. LiDAR‐derived ditch volume measurements are summarized in Table S4, while estimated years required to reach different blockage thresholds based on Total Station and LiDAR surveys are reported in Tables S5 and S6, respectively. Supplemental Figures S1–S5 illustrate experimental layout, instrumentation setup, representative LiDAR outputs, and field conditions, providing methodological context and supporting interpretation of site‐specific processes.
