# Supplementary material for: Effectiveness of narrow vegetative filter strips in regulating sediment deposition in floodplain agricultural drainage ditches
Source: J Environ Qual. 2026 Oct 8;55(5):e70280. doi: 10.1002/jeq2.70280 (PMC13650171; doi:10.1002/jeq2.70280)
Supplement: Supplementary file 1 [file JEQ2-55-0-s001.docx]

Supplemental Table S1. Yearly sediment deposition volume (m³) during the experimental period.

| Site name | VFS | Sediment deposition volume (m^3^) | | | |
| --- | --- | --- | --- | --- | --- |
|  | width (m) | 2020.6 - 2021.6 | 2021.6 - 2022.6 | 2022.6 - 2023.6 | Average |
| YAM | 0 | 104.8 | 117.1 | 45.2 | 89.0 |
|  | 2 | 87.5 | 164.9 | 29.4 | 93.9 |
|  | 4 | 157.6 | 52.9 | -58.0* | 50.8 |
| STC | 0 | 97.5 | 205.5 | 116.4 | 139.8 |
|  | 2 | 88.0 | 148.5 | 83.9 | 106.8 |
|  | 4 | 84.2 | 138.0 | 88.6 | 103.6 |
| BDF | 0 | Insufficient | 181.7 | 136.7 | 159.2 |
|  | 2 | profile | 129.9 | 79.0 | 104.4 |
|  | 4 | numbers | 80.0 | 160.0 | 120.0 |
| Average | 0 | 101.6 | 168.1 | 99.4 | 129.4 |
|  | 2 | 87.7 | 147.7 | 64.1 | 101.7 |
|  | 4 | 120.9 | 90.3 | 124.3 | 91.5 |
|  | all | 103.3 | 135.4 | 75.7 | 107.5 |

Supplemental Table S2. Standardized deposition per ditch length (m³/m)

| Site name | VFS | Standardized deposition (m^3^/m) | | | | |
| --- | --- | --- | --- | --- | --- | --- |
|  | width (m) | 2020.6 - 2021.6 | 2021.6 - 2022.6 | 2022.6 - 2023.6 | Average | |
| YAM | 0 | 0.202 | 0.225 | 0.087 | 0.171 | |
|  | 2 | 0.168 | 0.317 | 0.057 | 0.181 | |
|  | 4 | 0.303 | 0.102 | -0.112 | 0.098 | |
| STC | 0 | 0.188 | 0.395 | 0.224 | 0.269 | |
|  | 2 | 0.169 | 0.286 | 0.161 | 0.205 | |
|  | 4 | 0.162 | 0.265 | 0.17 | 0.199 | |
| BDF | 0 | Insufficient | 0.349 | 0.263 | 0.306 | |
|  | 2 | profile | 0.25 | 0.152 | 0.201 | |
|  | 4 | numbers | 0.154 | 0.308 | 0.231 | |
| Average | 0 | 0.195 | 0.323 | 0.191 | 0.249 | a |
|  | 2 | 0.169 | 0.284 | 0.123 | 0.196 | ab |
|  | 4 | 0.233 | 0.174 | 0.122 | 0.176 | b |

* Negative value indicates erosion happened during that period.

Yearly sediment deposition volumes and, on average, followed by the same letters are not significantly different at *p* = 0.10.

Supplemental Table 3. Sediment deposition volume during the growing season and non-growing season.

| VFS width (m) | Site name | Sediment deposition volume (m^3^) | | | | | | Volume changes rate | |
| --- | --- | --- | --- | --- | --- | --- | --- | --- | --- |
|  |  | Growing season | | Non-growing season | | Total | | between two periods | |
|  |  | (2021.6 - 2021.10) | | (2021.10 - 2022.6) | | (2021.6 - 2022.6) | | (%∆V) | |
| 0 | YAM | 57.7 | | 59.4 | | 117.1 | | 3.30% | |
|  | STC | 111.5 | | 94 | | 205.5 | | 29.67% | |
|  | BDF | 85.1 | | 96.6 | | 181.7 | | -90.50% | |
| 2 | YAM | 71.7 | | 93.1 | | 164.8 | | -15.86% | |
|  | STC | 88.7 | | 59.8 | | 148.5 | | -32.69% | |
|  | BDF | 86.5 | | 43.4 | | 129.9 | | 15.29% | |
| 4 | YAM | 48.3 | | 4.6 | | 52.9  138 | | -90.50% | |
|  | STC | 64.1 | | 73.9 | |  |  | 15.30% | |
|  | BDF | 38.7 | | 41.3 | | 80 | | 6.70% | |
| average | 0 | 84.8 | a | 83.3 | a | 168.1 | a | | -1.70% |
|  | 2 | 82.3 | a | 65.4 | a | 147.7 | a | | -20.50% |
|  | 4 | 50.4 | b | 39.9 | b | 90.3 | b | | -20.70% |

Sediment deposition volumes followed by the same letters are not significantly different at *p* = 0.10.

Supplemental Table S4. Ditch Volume Data by LiDAR.

| Site name | VFS width (m) | Ditch length (m) | Ditch volume (m^3)^ | | |
| --- | --- | --- | --- | --- | --- |
|  |  |  | 2019.11 | 2020.10 | 2021.10 |
| YAM | 0 | 607.5 |  | 1025.869 | 1377.816 |
|  | 2 | 619 | 850.5235 | 823.1142 | 391.0219 |
|  | 4 | 588 | 1212.448 | 846.3251 | 555.888 |
| STC | 0 | 818 | 1135.095 | 301.4201 | 310.3253 |
|  | 2 | 797.5 | 1068.157 | 499.731 | 443.5798 |
|  | 4 | 832.5 | 1799.316 | 1445.474 | 817.0651 |
| BDF | 0 | 1027.5 | 2786.044 | 1467.355 | 1481.924 |
|  | 2 | 1013.5 | 2406.043 | 1708.227 | 1299.664 |
|  | 4 | 1008 | 2235.382 | 1504.888 | 1223.949 |

Supplemental Table S5. Estimated years for blockage by Total Station surveys.

| Site name | VFS width (m) | Blockage Percentage | | | | |
| --- | --- | --- | --- | --- | --- | --- |
|  |  | 60% | 70% | 80% | 90% | 100% |
| YAM | 0 | 5.76 | 6.72 | 7.68 | 8.63 | 9.59 |
|  | 2 | 3.89 | 4.54 | 5.19 | 5.83 | 6.48 |
|  | 4 | 11.29 | 13.17 | 15.05 | 16.93 | 18.81 |
| STC | 0 | 3.92 | 4.57 | 5.22 | 5.87 | 6.53 |
|  | 2 | 4.89 | 5.70 | 6.52 | 7.33 | 8.15 |
|  | 4 | 4.33 | 5.05 | 5.77 | 6.50 | 7.22 |
| BDF | 0 | 7.09 | 8.27 | 9.45 | 10.64 | 11.82 |
|  | 2 | 8.99 | 10.49 | 11.98 | 13.48 | 14.98 |
|  | 4 | 9.60 | 11.20 | 12.80 | 14.40 | 16.00 |

Supplemental Table S6. Estimated years for blockage by LiDAR surveys.

| Site name | VFS width (m) | Blockage Percentage | | | | |
| --- | --- | --- | --- | --- | --- | --- |
|  |  | 60% | 70% | 80% | 90% | 100% |
| YAM | 0 |  |  |  |  |  |
|  | 2 | 2.15 | 2.51 | 2.87 | 3.22 | 3.58 |
|  | 4 | 1.55 | 1.80 | 2.06 | 2.32 | 2.58 |
| STC | 0 | 0.44 | 0.51 | 0.58 | 0.66 | 0.73 |
|  | 2 | 0.96 | 1.12 | 1.28 | 1.44 | 1.60 |
|  | 4 | 1.77 | 2.06 | 2.35 | 2.65 | 2.94 |
| BDF | 0 | 1.35 | 1.58 | 1.80 | 2.03 | 2.25 |
|  | 2 | 1.85 | 2.16 | 2.47 | 2.78 | 3.09 |
|  | 4 | 1.79 | 2.08 | 2.38 | 2.68 | 2.98 |

Supplemental Figure S1. Configuration of the vegetative filter strips (VFS) across three experimental sites, showing the arrangement of 2-meter and 4-meter-wide strips on both sides of the ditches.

Supplemental Figure S2. (a) LiDAR sensor and its platform UAV; (b) the GNSS-base station setup.

Supplemental Figure S3. Raw LiDAR data for YAM 4-m VFS ditch in 2020.

Supplemental Figure S4. In-ditch vegetation in June 2023 (the ditch with 4-m VFS at YAM).

Supplemental Figure S5. Beavers nest in the upstream of the ditch with a 2-m VFS at BDF.
